# Significance of anger suppression and preoccupied attachment in social anxiety disorder: a cross-sectional study

**DOI:** 10.1186/s12888-021-03098-1

**Published:** 2021-02-22

**Authors:** Rupert Conrad, Andreas J. Forstner, Man-Long Chung, Martin Mücke, Franziska Geiser, Johannes Schumacher, Friederike Carnehl

**Affiliations:** 1 Department of Psychosomatic Medicine and Psychotherapy, University Hospital Bonn, Venusberg Campus 1, 53127 Bonn, Germany; 2 Center for Human Genetics, University of Marburg, Baldingerstraße, 35033 Marburg, Germany; 3 Institute of Human Genetics, University of Bonn, Venusberg Campus 1, 53127 Bonn, Germany; 4 Center for Rare Diseases Bonn (ZSEB), University Hospital Bonn, Venusberg Campus 1, 53127 Bonn, Germany

**Keywords:** Social anxiety disorder, Attachment style, Mediation, Anger suppression, Anger expression, Preoccupied attachment

## Abstract

**Background:**

There is evidence for the relevance of attachment style and anger expression for the manifestation of social anxiety disorder (SAD).

**Method:**

In a cross-sectional study 321 individuals with social anxiety disorder (41% men, age 38.8 ± 13.9) were compared with 94 healthy controls (37% men, age 35.8 ± 15.1) on several questionnaires (Attachment Styles Questionnaire, State Trait Anger Inventory, Social Phobia Inventory, Beck Depression Inventory).

**Results:**

Individuals with SAD showed moderate-sized reduced levels of secure and large-sized increased levels of fearful and preoccupied attachment style compared to healthy controls (all *p* < 0.001) as well as small-sized increased levels of trait anger (*p* = 0.03) and moderate-sized increased levels of anger-in (*p* < 0.001). Attachment style and anger regulation could predict 21% (R^2^ = 0.21, p < 0.001) of the extent of social anxiety (SPIN) in SAD; *secure* (β = − 0.196, *p* < 0.01) and *preoccupied attachment style* (β = 0.117, *p* < 0.05), as well as *anger-in* (β = 0.199, *p* < 0.01) were significant cross-sectional predictors. Further analysis revealed that the relationship between *preoccupied attachment* and social anxiety is partially mediated by *anger-in*.

**Conclusion:**

Study findings confirm the relevance of preoccupied attachment style and anger suppression for social anxiety. Disentangling the role of anger regulation in early attachment patterns has significant therapeutic implications in SAD.

## Background

Social anxiety disorder as defined by the Diagnostic and Statistic Manual of Mental Disorders in its latest editions (DSM-IV [1], DSM-5 [2]) is characterized by a persistent fear of one or more social or performance situations in which the person is exposed to unfamiliar people or to possible scrutiny by others. The individual fears that he or she will act in a way (or show anxiety symptoms) that will be embarrassing and humiliating. This insecurity in social situations underlie specific inner representations of others and stable behavioral patterns, defined as attachment behavior [3]. By means of this behavior a child manages feelings of stress or fear through the regulation of proximity to a caregiver.

Specifically, socially anxious individuals often show less secure attachment, which is characterized by a positive self-image and a positive image of others. Instead they show an insecure attachment, which distinguishes between three different attachment styles [3]. First, the preoccupied attachment style, which is defined by a negative view of self and a positive view of others. People with preoccupied attachment style are characterized by a dependent interpersonal style as well as a deep-rooted lack of selfworth. Second, the fearful attachment style, which is defined by a negative view of self and others and leads to an avoidance of close relationships as a means to protect oneself against anticipated rejection. Finally, the dismissing attachment style, which is defined by a positive view of self and a negative view of others and goes along with the avoidance of close relationships as a means to safeguard oneself against being disappointed by others [4]. Regarding individuals suffering from SAD, who tend to worry about being rejected and strongly desire to feel near to others, there is empirical evidence for an increased frequency of the preoccupied attachment style [5, 6]. Given the importance of early experiences to the development of emotion regulation abilities, caregiver-child attachment relationships may be a significant precursor to the onset of emotion regulation difficulties [7]. Thus, in insecure attachment relationships with inconsistent emotional availability caregivers are not readily available to soothe their children when distressed, which impacts children’s emotion regulation abilities. Against this backdrop there is growing evidence which suggests that attachment theory can be viewed as a theory of affect regulation, given that the regulation of emotions in the caregiver-child relationships lays the foundations for the self-regulation of emotions [8, 9]. In line with this assumption several studies found a deficiency in the ability to regulate emotions in individuals with SAD [10–13]. Our study primarily addressed specific difficulties in the experience and expression of anger as defined by the state-trait anger expression model [14]. In the experience of anger it differentiates between state anger as the current experience of angry feelings ranging from mild annoyance to fury, and trait anger as a personality-based disposition to feel anger across a wide range of situations. With regard to anger expression it distinguishes between a behavioral tendency to direct anger inwards and conceal angry feelings, a tendency to direct anger outwards, and a tendency to control anger by avoiding annoyance or pacifying oneself through cognitive strategies. There is evidence that the anticipated fear of negative evaluation and rejection in SAD provokes anger across a wide range of situations corresponding to increased trait anger [15, 14]. The increased experience of anger poses a great predicament for individuals with SAD, as the expression may increase the real or perceived threat of further negative evaluation [16]. Therefore anger may evoke anxiety and is suppressed in order to reduce anxiety. In keeping with this assumption previous studies found higher levels of suppressed anger in SAD [15–17].

From a therapeutic perspective anger suppression has been identified as an important predictor for worse therapeutical outcome in cognitive behavioral therapy of SAD [15].

Against the backdrop of the above outlined close association of attachment style and emotion regulation ability it is important to gain further insight into the relationship between the experience and expression of anger and insecure attachment. Within this framework we assumed that difficulties in anger regulation in SAD basically derive from preoccupied attachment style and partially mediate the association between this attachment pattern and social anxiety [18, 7]. No previous study investigated the relationship between anger expression and attachment in SAD.

Our study tested the following novel hypotheses: Individuals diagnosed with SAD in comparison to the control group are predicted to have moderate effect size reduced rates of secure attachment (I) and large effect size increased rates of preoccupied attachment style (II). With regard to anger, individuals with SAD show small effect size increased rates of trait-anger (III) and moderate effect size increased rates of anger suppression (IV). Furthermore, in linear regression analysis with attachment styles and anger dimensions as independent variables secure attachment style, preoccupied attachment style and anger suppression are significant cross-sectional predictors for the intensity of social anxiety (V). As outlined above mirroring and responding to the child’s affective state by the caregiver essentially constitutes the child’s attachment style, on which the child’s emotion regulation ability is fundamentally based. Within this framework we hypothesize that anger suppression partially mediates the association between preoccupied attachment and social anxiety (VI).

## Methods

Recruitment of the SAD sample was organized within the framework of the research project “Social Phobia Research”. The project is based on a cooperation between the Institute of Human Genetics and the Department of Psychosomatic Medicine and Psychotherapy at Bonn University Hospital as well as the Centre of Human Genetics at the University of Marburg, Germany [19–21]. The study participants and controls were recruited between January 2013 and June 2019 via advertisements, radio, television, newspaper articles or clinical services. The ethics committee of the University of Bonn approved the study (No. 222/12) and all participants signed informed consent. Inclusion in the study group required the confirmation of a current diagnosis of SAD by the DSM-IV version of the Structured Clinical Interview for Axis I disorders (SCID-I) and a score of ≥25 in the Social Phobia Inventory (SPIN) [22, 23]. All participants had to be 18 years or above. Exclusion criteria were bipolar disorder and schizophrenia, insufficient German language skills or somatic (e.g. acute pain) and/or mental difficulties (e.g. acute substance-related intoxication, acute suicidality) in completing the questionnaires.

Inclusion criteria for the control group were age ≥ 18 and no current or lifetime mental disorder as confirmed by the Structured Clinical Interview for DSM-IV Axis I disorders. Exclusion criteria were insufficient German language skills to complete study questionnaires.

The sample comprised 321 participants with social anxiety disorder and 94 healthy control subjects. Table 1 presents sociodemographic and clinical characteristics.

**Table 1:** **Sociodemographic and clinical characteristics**

	SAD group	Control group	Test statistic (*p*-value)
	*n* = 321	*n* = 94	
Characteristics:	*n* (%)	*n* (%)	
Gender			
Female	189 (58.9)	59 (62.8)	χ^2^ = 0.457
Male	132 (41.1)	35 (37.2)	(0.499)
Age (in years)			
*M*	38.83	35.76	*t* = 1.85
(*SD*)	(13.85)	(15.14)	(0.064)
Formal education			
Below high school	103 (32.1)	7 (7.4)	χ^2^ = 23.04
High school	120 (37.4)	51 (54.3)	(< 0.001) ***
College level or higher	98 (30.5)	36 (38.3)	
Depression (BDI)			
*M*	21.35	3.89	*t* = 21.08
(*SD*)	(11.09)	(5.33)	(< 0.001) ***
Social Anxiety (SPIN)			
*M*	42.79	7.68	*t* = 45.22
(*SD*)	(9.36)	(5.57)	(< 0.001) ***

Note: *** *p* ≤ 0.001, two-tailed

### Measures

#### Demographics

Age, sex, level of education, ethnicity, partnership status, treatment for mental disorders, and somatic and mental disorders in family members were assessed by a self-report questionnaire. We used age and sex as covariates in our analyses.

#### Diagnoses

Trained and supervised expert interviewers assigned diagnoses by means of the German version of the SCID-I for DSM-IV [24, 25]. This semi-structured interview shows high reliability and validity [24].

#### Depression

Depression was assessed with the Beck Depression Inventory (BDI) [26] consists of 21-items scored on a 4-point Likert scale. Higher total scores indicate more severe depressive symptoms. The BDI is a highly reliable and valid psychometric instrument showing a Cronbach’s alpha of 0.93 in our study.

#### Severity of social anxiety symptoms

In addition to the SAD Diagnosis, the Social Phobia Inventory (SPIN) [22, 23] was used to measure the severity of SAD symptoms. The self-report inventory consists of 17 items with a sum score ranging between 0 and 68, higher scores indicate more severe social anxiety symptoms. The SPIN is a highly economical screening instrument and shows good psychometric properties [23]. The Cronbach’s alpha of 0.96 in the current study demonstrated excellent internal consistency.

#### Attachment style

This was measured by the Attachment Style Questionnaire (ASQ) [27]. The different attachment styles *secure*, *preoccupied, fearful* and *dismissing* are measured with 22 items on a 5-point Likert scale. Previous studies showed high construct validity and reliability [28]. Higher scores indicate higher manifestation of the respective attachment style, e.g. a higher score on the scale preoccupied attachment means a greater tendency to worry about being rejected and to feel dependent on others. A higher score on the fearful attachment style means a negative view of self and others and a greater tendency to feel uncomfortable with emotional closeness. A higher score on the dismissing attachment style means a positive view of self and a negative view of others, which is associated with a greater desire of independence and the avoidance of any attachment. In this study the ASQ demonstrated good internal reliability, with Cronbach’s alpha of 0.80 (secure), 0.87 (fearful), 0.81 (preoccupied) and 0.70 (dismissing).

#### Anger

Anger was assessed using the State-Trait Anger Expression Inventory (STAXI) [14]. State and trait anger are measured as well as three dimensions of anger expression: *anger-in*, *anger-out* and *anger-control*. The questionnaire comprises 44 items scored on a 4-point Likert scale, higher scores indicate higher manifestation of anger state/trait or expression style, e.g. higher scores on the trait-anger scale means a greater disposition to feel anger across a wide range of situations, whereas state anger refers to the current experience of angry feelings along a continuum from little anger through mild annoyance to highly emotionally charged states such as fury and rage. Regarding anger expression styles higher scores on the anger-in scale mean a greater tendency to hold in and suppress angry feelings, whereas higher scores on the anger-out scale mean a higher tendency to express angry feelings towards other persons or objects in the environment. Higher scores on anger-control mean a greater tendency to avoid anger in the first place by avoiding annoyance or to pacify oneself as early as possible through cognitive strategies.

The questionnaire demonstrated good psychometric properties, with Cronbach’s alpha of 0.93 (state anger), 0.89 (trait anger), 0.86 (anger-out), 0.89 (anger-in), 0.83 (anger control).

### Statistical analyses

Descriptive statistics were presented for sociodemographic and clinical data. Group differences were analysed by F-tests and chi-square tests .

The group effect was examined by analysis of covariance (ANCOVA) with age, sex and depression as covariates. Furthermore, Pearson correlations were calculated for intercorrelations between study variables. Stepwise regression analysis was used to predict the degree of social anxiety in our study group by means of attachment style and anger. *p* values < 0.05 were evaluated as statistically significant, p values < 0.10 ≥ 0.05 as a statistical trend [29]. To evaluate the effect size of differences we calculated Cohen’s d, effect sizes were interpreted according to recommendations as small (0.2 to < 0.5), moderate (0.5 to < 0.8), or large (≥0.8).

For the exploratory mediator analysis we used the PROCESS macro [30] in SPSS (version 24.0.0.0).

## Results

### Sociodemographic and clinical characteristics

The study and control group showed no significant differences with regard to sex and age. The level of education differed significantly with a higher education level for the control group. Obviously, depressive symptomatology as measured by the BDI was significantly increased in the SAD sample. Regarding comorbidity with substance-related disorders 22 individuals (6,9%) showed alcohol dependence and 67 alcohol abuse (20,9%); concerning other substance-related disorders 6 subjects (1,9%) fulfilled criteria of dependence and 2 of substance abuse (0,6%).

### Attachment style and SAD

**Fig. 1:**
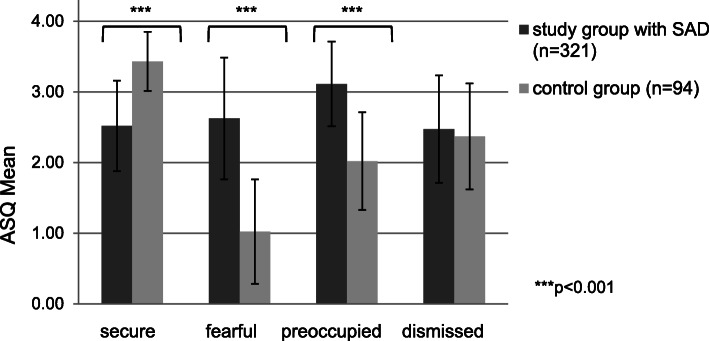
Results for ASQ (ANCOVA, covariates age, sex, depression)

The first ANCOVAs (Fig. 1) revealed that participants with SAD compared to healthy controls showed significantly moderate-sized lower levels of secure attachment style (*F* = 41.92, *p <* 0.001, d = 0.64) and scored higher on preoccupied (*F* = 100.28, *p* < 0.001, d = 0.99) as well as fearful attachment style (*F* = 85.74, *p* < 0.001, d = 0.91) by a large effect size. These findings confirm our hypotheses (I) and (II). Additionally we found significant differences concerning fearful attachment style.

### Trait anger, anger-in and SAD

**Fig. 2:**
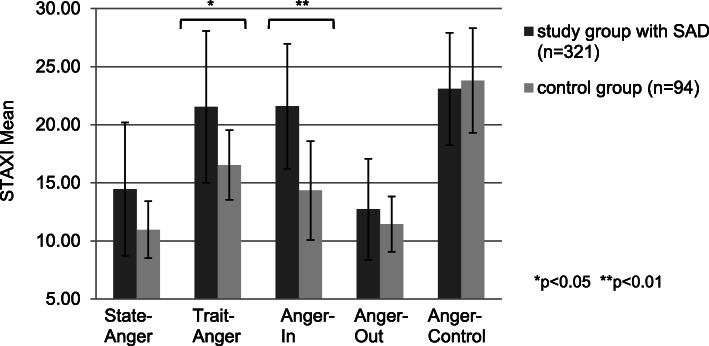
Results for STAXI (ANCOVA, covariates age, sex, depression)

In line with our hypotheses (III) and (IV), further ANCOVAs (Fig. 2) revealed small-sized increased levels of trait anger for socially anxious indviduals compared to the control group (*F* = 4.718, *p* = 0.030, d = 0.21). Concerning anger-in the differences were significant confirming that socially anxious individuals report moderate-sized higher levels of anger-in (*F* = 35.687, *p* < 0.001, d = 0.59).

### Intercorrelations between study variables

In the next step of analysis we calculated intercorrelations between our study variables in both groups (Table 2 and Table 3).

**Table 2:** Intercorrelation matrix SAD

	1.	2.	3.	4.	5.	6.	7.	8.	9.	10.	11.
**1. SPIN**	1										
**2. ASQ-secure**	−.32**	1									
**3. ASQ-fearful**	.32**	−.55**	1								
**4. ASQ-preoccupied**	.21**	−.06	.26**	1							
**5. ASQ-dismissing**	.05	−.29**	.21**	−.20**	1						
**6. STAXI-State anger**	.16**	−.28**	.28**	.04	.02	1					
**7. STAXI-Trait anger**	.19**	−.28**	.28**	.13*	.10	.41**	1				
**8. STAXI-Anger-in**	.37**	−.36**	.44**	.16**	.14*	.32**	.39**	1			
**9. STAXI-Anger-out**	.12*	−.21**	.21**	.10	.10	.34**	.70**	.28**	1		
**10. STAXI-Anger-control**	.14*	.05	.12*	.03	−.02	−.16**	−.38**	.24**	−.49**	1	
**11. BDI**	.39**	−.39**	.38**	.21**	.04	.44**	.32**	.36**	.29**	−.10	1

Note: * *p* ≤ 0.05, ** *p* ≤ 0.01

**Table 3:** Intercorrelation matrix Controls

	1.	2.	3.	4.	5.	6.	7.	8.	9.	10.	11.
**1. SPIN**	1										
**2. ASQ-secure**	.13	1									
**3. ASQ-fearful**	.28**	−.16	1								
**4. ASQ-preoccupied**	.46**	−.08	.41**	1							
**5. ASQ-dismissing**	−.03	.01	.20	−.09*	1						
**6. STAXI-State anger**	.18	−.20	.39**	.33**	.03	1					
**7. STAXI-Trait anger**	.30**	−.01	.22*	.27**	−.05	.04	1				
**8. STAXI-Anger-in**	.15	−.31**	.29**	.11	.21*	.10	.24*	1			
**9. STAXI-Anger-out**	.13	.14	.16	.19	−.12	.04	.51**	.02	1		
**10. STAXI-Anger-control**	−.17	.02	−.13	−.10	.21*	−.15	−.22*	.18*	−.40**	1	
**11. BDI**	.34**	−.17	.47**	.37**	.13	.66**	.16	.13	.03	−.10	1

Note: * *p* ≤ 0.05, ** *p* ≤ 0.01

Regarding individuals with SAD social anxiety (SPIN) showed highly significant correlations (*p* < 0.01) with secure, preoccupied and fearful attachment style as well as state anger, trait anger, anger-in and depression. In controls social anxiety showed significant associations with preoccupied as well as fearful attachment style, trait anger and depression.

### Attachment style and anger as cross-sectional predictors for SAD

The stepwise regression analysis tested our hypothesis (V) that secure attachment style, preoccupied attachment style and anger-in serve as significant cross-sectional predictors for social anxiety taking sex and age into account. The results can be seen in Table 4.

**Table 4:** Stepwise regression analysis, dependent variable SPIN, predictors sex, age (step 1), ASQ, STAXI (step 2)

Stepwise regression analysis			
	predictors	Social Anxiety (SPIN)	
		*β*	*R*^2^
step 1	sex	.064	
	age	−.006	
			.004
step 2	secure attachment	−.196**	
	preoccupied attachment	.117*	
	anger-in	.199**	
			.213***

Note: N = 321; *p < .05,** *p* < 0.01,

Altogether cross-sectional predictors explained about 21.3% of variance in social anxiety as measured by SPIN. Secure attachment (β = −.196, *p* < 0.01) and preoccupied attachment (β = .117, *p* < 0.05) styles were significant predictors for SAD. Furthermore, anger-in (β = .199, *p* < 0.01) significantly predicted social anxiety.

### Mediation analysis

**Fig. 3:**
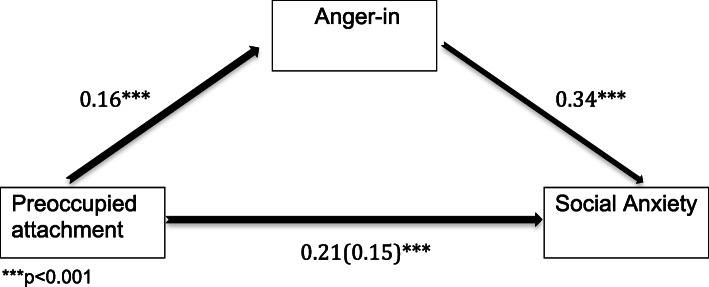
Mediation analysis for preoccupied attachment (ASQ) and social anxiety (SPIN) with anger-in (STAXI) as mediator

The analysis confirmed our hypothesis VI and showed that the relationship between preoccupied attachment style and intensity of social anxiety is partially mediated by anger-in (Fig. 3), with a standardized total effect between preoccupied attachment and social anxiety of 0.21 and an indirect effect of 0.06 (95% CI 0.02–0.09).

## Discussion

In our study we aimed at investigating the significance of attachment style and anger in individuals with SAD compared to healthy controls.

### Attachment

Our hypotheses of moderate-sized reduction of secure attachment and large-sized increase of preoccupied attachment in socially anxious individuals were confirmed. These findings are in line with empirical results in previous studies [5, 6, 31, 32]. We did not hypothesize the significant difference with regard to fearful attachment style, which also showed a large effect size.

The preoccupied attachment style is often correlated with the experience of inconsistent and insensitive parenting [33] and an inner assumption that the own unworthiness explains any lack of love from the caregiver. In a previous study [5] individuals with social anxiety and preoccupied attachment felt less well-being in personal relationships, less alacrity to trust others and greater anxiety of rejection compared to the control sample. Particulary, preoccupied attached individuals without a partner experience severe social anxiety and depression [34]. The large-sized increase in fearful attachment may be explained by a generally increased prevalence of traumatic experiences and adverse childhood events in the SAD sample compared to healthy controls. Previous studies showed an increase of this attachment style, which goes along with a negative view of others and the avoidance of close relationships, in traumatized samples [35, 36].

### Anger

Our hypotheses of a small-sized increase of trait anger as well as moderate-sized increase of anger suppression were confirmed. These findings correspond to a previous study [15], which reported poorer overall skills to express anger in socially anxious individuals. This might be explained by the fact that the expression of anger may increase the threat of negative evaluation. Consequently, suppression of anger may be utilized to reduce this anxiety. Since individuals with SAD are likely to interpret ambiguous social stimuli as threatening and identify neutral events as dangerous, rejection is more salient to them [16]. This might be particularly problematic as feared rejection is a potent antecedent of anger for individuals with social anxiety [16] rendering coping with anger particularly challenging [15].

### Anger and attachment for social phobia

The results of our regression analysis show that both, attachment style and anger suppression serve as significant cross-sectional predictors for social anxiety. The mediation model suggests that the relationship between preoccupied attachement style and the degree of social anxiety is partially mediated by anger suppression. How can this relationship be explained?

A highly relevant factor underlying anger suppression is attachment style [37]. Primary attachment is closely intertwined with caregivers’ emotional involvement and resonance thereby furthering a child’s mentalization and emotional self-regulation [9, 7]. Contigently mirroring and responding to the child’s affective state constitutes secure attachment as well as the child’s emotion regulation ability.

Cassidy (1994) specifies that preoccupied attached children are likely to have parents who do not help to regulate their distress [38]. The child’s negative emotionality often fulfills the parents’ own attachment needs, showing that the child wants to stay close. Insecurely attached children apply emotion regulation strategies like shifting attention away from the caregiver or suppressing the elicited emotion [7]. Thus, the elevated suppression of anger found in our study could mirror the experience of inconsistent early attachment to the caregiver. With regard to social anxiety the preoccupied attachment style could also explain for higher fearfulness in response to relatively benign stimuli, since the expression of fear increases caregivers’ attention [38] thereby reassuring the mutual bond.

### Implications

Study results could have implications for the treatment of socially anxious patients, however the cross-sectional study design demands the confirmation of our findings in future longitudinal studies. Since attachment styles significantly predicted the severity of social anxiety in our study, they should be assessed and taken into consideration in diagnosis and therapy. Previous studies indicate on the one hand that attachment patterns are relevant predictors of psychotherapeutic outcome in social anxiety disorder [39], on the other that cognitive-behavioral therapy may change these patterns [40]. In addition to that, insecure attachment is generally associated with greater rejection of health care providers, less treatment compliance and self-disclosure [41] highlighting the importance of addressing this issue in the therapeutical context. By means of establishing a positive interpersonal relationship in therapy, the patients can modify or extend their internalized working models of attachment. With regard to anger our findings emphasize the importance of healthy anger management for SAD patients. Anger supression partially mediates early insecure attachment patterns thereby complicating the establishment of a therapeutical alliance and increasing the likelihood of non-adherence [42–44]. Furthermore, anger may greatly exhaust individuals’ time and energy [16]. Energy that is lacking when it comes to strengthening or deepening relationships. Psychotherapy should increase the patients’ awareness for their early attachment experiences and the way they cope with anger. Encouraging socially anxious patients to try out more adaptive and flexible styles of anger expression may strengthen the therapeutic relationship and help to establish more secure attachment patterns.

### Strengths and limitations

Our findings should be interpreted with careful consideration of the following methodological strengths and weaknesses. A strength of our study is the heterogeneity of our sample. Our cohort comprised clinical and non-clinical individuals and was recruited from various sources which contributes to the external validity of the present findings. Furthermore, our results rely on a big SAD-cohort and a control group with the gold standard of diagnoses assigned by SCID-I.

Despite these strengths, the present study shows several limitations. First, we conducted a cross-sectional study which does not allow for a causal interpretations of our findings. Second, we used self-rating instruments assessing for attachment style and anger which can be influenced by reporting biases. Third, there was no standardized assessment of personality disorders by SCID-II.

## Conclusions

In summary, individuals with SAD show less secure and more preoccupied attachment as well as higher scores on trait anger and anger suppression. Preoccupied attachment and anger suppression are significant cross-sectional predictors for social anxiety; anger suppression partially mediates preoccupied attachment and social anxiety. An integrative focus on the association of insecure attachment style and anger suppression in SAD may facilitate therapeutic communication and optimize clinical outcomes.

## Data Availability

Data will be available from the corresponding author (RC) upon request after publication.
